# Questionnaire survey on cervical cancer screening and HPV awareness among patients at a local cancer center in Japan

**DOI:** 10.1186/s12905-024-03256-z

**Published:** 2024-07-16

**Authors:** Kazuto Nakamura, Keiko Kigure, Toshio Nishimura, Soichi Yamashita

**Affiliations:** Department of Gynecology, Gunma Prefectural Cancer Center, 617-1 Takabayashi-nishi, Ota, Japan

**Keywords:** Cervical cancer, Screening, HPV, Active recruitment strategy

## Abstract

**Background:**

The incidence of cervical intraepithelial neoplasia is increasing in Japan. Although human papillomavirus (HPV) vaccination and cancer screening are crucial in preventing cancer-related mortality, the cervical cancer screening rate in Japan was only approximately 43.6% in 2022. This study aimed to conduct an epidemiological analysis of cervical cancer by collecting data from individual patients.

**Methods:**

A questionnaire survey was administered to patients who visited our hospital between January 2017 and July 2023 owing to abnormal cervical cytological findings or a cancer diagnosis. Patients answered questions regarding their history of cervical cancer screening as well as their knowledge of HPV and cervical cancer.

**Results:**

During the study period, 471 patients participated in the survey, with 35 declining to participate. Patients with Stage 1b1–4b primarily sought medical attention due to self-reported symptoms (*P* < 0.001); however, they were less likely to have undergone cervical cancer screening (*P* < 0.001). Additionally, older patients were less likely to be aware of the association of HPV with cervical and other cancers. Notably, 28 of the 129 patients with stage 1b1–4b cancer underwent cervical cancer screening within 2 years. The tumor location within the endocervical canal emerged as a significant factor contributing to the difficulty for an accurate diagnosis of precancerous or cervical cancer during cervical screening. Furthermore, non- squamous cell carcinoma (SCC) histology was another possible factor.

**Conclusions:**

Our findings suggest the need to widely disseminate information regarding the significance of cancer screening to increase cancer screening rates. Moreover, establishing strategies for improving the accuracy of detecting lesions during screening for non-SCC and endocervical canal tumors is crucial.

**Supplementary Information:**

The online version contains supplementary material available at 10.1186/s12905-024-03256-z.

## Introduction

In 2020, the World Health Organization issued a statement to eliminate cervical cancer using human papillomavirus (HPV) vaccination, highly accurate cancer screening, and effective treatment of pre-invasive and invasive cancers [1]. The incidence of cervical cancer has been increasing since 2000 in Japan [2], which is in contrast to that in other developed countries. HPV vaccination and cervical cancer screening crucially contribute toward the prevention of cancer-related mortality. The Japanese government started providing subsidy for HPV vaccination of girls aged 13–16 years in 2010. However, in June 2013, HPV vaccination was suspended because of repeated media reports regarding its adverse effects [3], resulting in a decrease in vaccine coverage from 68.9% for individuals born in 1999 to 0.2% for individuals born in 2003 [4, 5]. As of 2022, the average HPV vaccination rate for women aged 25–30 years is between 53.4 and 78.5%, but it is below 1% for women aged < 25 years. After a 9-year suspension, the Ministry of Health, Labor, and Welfare of Japan announced the resumption of HPV vaccination [6], which was reinstated in local municipalities in April 2022. Contrastingly, Australia, which is among the highly advanced countries with early and extensive HPV vaccination coverage, has achieved a clear reduction in the incidence of cervical neoplasia [7]. The efficacy of HPV vaccination has also been demonstrated in Japan [8]. However, despite numerous efforts toward the resumption of HPV vaccination, a longer period of time may be required to achieve a significant decrease in the incidence of cervical cancer through extensive countrywide vaccine coverage in Japan.

Additionally, cervical cancer screening for the detection of pre-cancerous lesions has been shown to reduce the occurrence of invasive cancer in the USA [9] and Japan [10]. In Japan, eligible women aged 20–69 years receive an invitation letter for cervical cancer screening from local governments every 2 years as per the Japan cervical cancer screening guidelines [11]. Therefore, most women have an opportunity to undergo screening. Moreover, some women undergo either workplace-provided or opportunistic screening. Despite the adoption of a robust screening program in the 1980s, the screening rate in Japan has been substantially lower than that in Western countries. According to OECD Health Statistics 2023, 42.4% of women aged 20–69 years in Japan underwent a Pap smear test, which is lower than the > 70% rate among targeted women in Sweden, the USA, and the UK [12]. Various factors may impede screening, including cost, busy daily lifestyle, health illiteracy, and inadequate information regarding cervical cancer.

Taken together, increasing the screening rate in Japan is important. Therefore, this study aimed to conduct a questionnaire survey of patients who visited our hospital with suspected cervical neoplasia or a cancer diagnosis. The study objectives were as follows: (i) to understand the demographic characteristics of patients, (ii) to assess patients’ knowledge regarding cervical cancer and HPV, (iii) to identify weaknesses in the current strategy for cancer screening, and (iv) to inform strategic improvements for increasing the screening rate.

## Methods

A questionnaire survey was administered to patients who visited our hospital between January 2017 and December 2023 owing to abnormal cervical cytological findings or a cancer diagnosis. In Japan, individuals can undergo cervical cancer screening using any of the following ways: organized screening for eligible individuals aged > 20 years at 2-year intervals, periodic health checkups provided in the workplace as a benefit package, and opportunistic screening sought individually. After the Bethesda system was applied for cervical cancer screening in our medical district, a cone-shaped brush was primarily used to obtain samples for liquid-based cytology.

Patients provided written informed consent and responses regarding their history of cervical cancer screening as well as knowledge of HPV and cervical cancer. The questionnaire produced for this study is presented in the supplementary file. This study was approved by the Ethics Committee of Gunma Prefectural Cancer Center (approval # 405–04064) and was conducted in accordance with relevant guidelines and regulations (Declaration of Helsinki). Responses were analyzed with respect to cervical cancer status using descriptive analysis methods, including the Kruskal–Wallis test, chi-square test, logistic analysis, and Fisher’s exact test. All statistical analyses were performed using EZR version 1.55 [13].

## Results

From January 2017 to December 2023, 471 patients were included in this study, whereas 35 individuals declined to participate. All patients were transferred to our hospital owing to abnormal cytological findings, suspected cancer, or a diagnosis of cervical cancer. At the initial visit, 68 patients exhibited no lesions. However, 79, 195, 38, and 91 patients had CIN1–2, CIN3- the International Federation of Gynecology and Obstetrics (FIGO 2018) Stage1a, Stage 1b1, and Stage 1b2–4b cervical cancer, respectively (Table 1). No patients aged 20–29 years were diagnosed with Stage 1b1 and Stage 1b2-4b cervical cancer. However, a distinct proportion of patients with advanced cervical cancer (Stage 1b2-4b) was observed in the older age group, with patients aged ≥ 50 years. Patients with Stage 1b1–4b primarily visited our hospital because of self-reported symptoms, including vaginal bleeding and abnormal discharge. Contrastingly, patients with no lesions and CIN1–2 and CIN3-Stage 1a were mainly referred following abnormal cytological results (*P* < 0.001). In contrast to no lesions and CIN1–2, patients with Stage 1b1 and Stage 1b2–4b cervical cancer had a longer interval of cancer screening or had never undergone it (*P* < 0.001). We then examined patients who had undergone cervical screening until this survey. In all age groups, most patients underwent cancer screening following invitation letters from local municipalities, followed by health-checkup programs provided in the workplace (Table 2). Subsequently, we examined the association between cervical lesions and age at first screening (Table 3). Notably, a significant proportion of patients in Stage 1b2–4b underwent cancer screening at the age of ≥ 50 years. Furthermore, we assessed all patients’ knowledge of cervical cancer and HPV. In all age groups, low awareness was noted regarding the peak incidence age of cervical cancer and the fact that smoking was a risk factor for cervical cancer (Table 4). Many patients were aware of the absence of clinical symptoms of early-stage cervical cancer and the effectiveness of screening during their hospital visit. Furthermore, older patients were less likely to recognize HPV as sexually transmitted and its association with cervical cancer. Moreover, most patients were unaware of the association of HPV with other cancers, including vulva, anal, and mesopharyngeal cancers. Notably, 28 of the 129 patients with Stage 1b1–4b cervical cancer underwent screening within 2 years. Furthermore, 60.7% of these patients had a tumor size of > 2 cm. Tumor location within the endocervical canal was the significant factor contributing to difficulties in diagnosing cervical cancer (*P* < 0.005), with non-SCC histology approaching statistically significant difference (*P* = 0.058) (Table 5).

**Table 1:** Patients characteristics at initial visit to our hospital

Initial diagnosis	No lesion	CIN1-2	CIN3-Stage1a	Stage 1b1	Stage Ib2-4b	*p* value
	*n*=68	*n*=79	*n*=195	*n*=38	*n*=91	
Age at visit to our hospital						*P*<0.001*
20-29 years old	2 (2.9%)	7 (8.9%)	25 (12.8%)	0 (0%)	0 (0%)	
30-39 years old	12 (17.6%)	20 (25.3%)	64 (32.8%)	8 (21.1%)	6 (6.6%)	
40-49 years old	29 (42.6%)	23 (29.1%)	73 (37.4%)	16 (42.1%)	20 (22.0%)	
≥ 50 years old	25 (36.8%)	29 (36.7%)	33 (16.9%)	14 (36.8%)	65 (71.4%)	
Reason of introduction to our hospital						*P*<0.001*
Abnormal cytology result	58 (85.3%)	68 (86.1%)	140 (71.8%)	13 (34.2%)	9 (9.9%)	
Self symptoms	4 (5.9%)	3 (3.8%)	24 (12.3%)	17 (44.7%)	71 (78.0%)	
Visit to a clinic for other reason	6 (8.8%)	8 (10.1%)	31 (15.9%)	8 (21.1%)	11 (12.1%)	
Interval of cervical cancer screening						*P*<0.001*
First screening	0 (0.0%)	3 (4.3%)	15 (7.7%)	1 (2.6%)	2 (2.2%)	
≦2 years	51 (75.0%)	65 (82.3%)	111 (56.9%)	13 (34.2%)	15 (16.5%)	
2<≤5 years	6 (8.8%)	6 (7.6%)	27 (13.8%)	5 (13.2%)	9 (9.9%)	
5<≤10 years	2 (2.9%)	1 (1.3%)	18 (9.2%)	6 (15.8%)	12 (13.2%)	
>10 years	4 (5.9%)	0 (0.0%)	6 (3.1%)	3 (7.9%)	14 (15.4%)	
Never screened	5 (7.4%)	4 (5.1%)	18 (9.2%)	10 (26.3%)	39 (42.9%)	

*Kruskal-Wallis test was used

**Table 2:** Reason of cervical screening in various age groups

Starting age of screening	20-29 years old	30-39 years old	40-49 years old	≥ 50 years old	*P*-value
	*n*=29	*n*=94	*n*=132	*n*=123	
Advice from mother or relatives	9 (31.0%)	8 (8.5%)	3 (2.3%)	5 (4.1%)	*P*<0.001*
Advice from friend	0 (0.0%)	3 (3.2%)	9 (6.8%)	11 (8.9%)	
Invitation from local screening programme	11 (37.9%)	47 (50.0%)	67 (50.8%)	55 (44.7%)	
Understanding of screening significance	0 (0.0%)	1 (1.1%)	5 (3.8%)	2 (1.6%)	
Health check programme in working place	2 (6.9%)	19 (20.2%)	32 (24.2%)	29 (23.6%)	
Other reason	7 (24.1%)	16 (17.0%)	16 (12.1%)	21 (17.1%)	

*Chi-squared test was used. Seven teen patients who underwent screening were excluded because of no answer about strating age

**Table 3:** Association between cervical lesion and starting age of cervical screening

Diagnosis	No lesion	CIN1-2	CIN3-Stage1a	Stage 1b1	Stage Ib2-4b	*P*-value
	*n*=60	*n*=73	*n*=163	*n*=27	*n*=46	
Starting age of screening						*P*<0.001*
20-29 years old	19 (31.7%)	28 (38.4%)	78 (47.9%)	10 (37.0%)	6 (13.0%)	
30-39 years old	16 (26.7%)	25 (34.2%)	60 (36.8%)	7 (25.9%)	13 (28.3%)	
40-49 years old	19 (31.7%)	15 (20.5%)	17 (10.4%)	6 (22.2%)	12 (26.1%)	
≥ 50 years old	6 (10.0%)	5 (6.8%)	8 (4.9%)	4 (14.8%)	15 (32.6%)	

*Kruskal-Wallis test was used. Twenty-six patients who underwent screening were excluded because of no answer about starting age

**Table 4:** Comprehesive understanding about cervical cancer and HPV

Age at visiting to our hospital	≤ 30 years	31-40 years*	41-50 years**	51-60 years	60 ≥ years	Odds ratio [95%CI]	*P*-value
	*n*=34	*n*=110	*n*=161	*n*=102	*n*=64		
Questions about cervical cancer							
Peak incidence rate is around 40 years old						1.10 [0.924-1.320]	*P*=0.237^#^
Yes	5 (14.7%)	23 (20.9%)	66 (41.0%)	22 (21.6%)	19 (29.7%)		
No	29 (85.3%)	87 (79.1%)	95 (59.0%)	80 (78.4%)	45 (70.3%)		
No clinical symptom in very early stage						0.895 [0.745-1.08]	*P*=0.24^#^
Yes	26 (76.5%)	86 (78.2%)	119 (73.9%)	74 (72.5%)	45 (70.3%)		
No	8 (23.5%)	24 (21.8%)	42 (26.1%)	28 (27.5)	19 (29.7%)		
Effectiveness of cervical cancer screening						0.859 [0.676-1.09]	*P*=0.212^#^
Yes	28 (82.4%)	96 (87.3%)	150 (93.2%)	85 (83.3%)	51 (79.7%)		
No	6 (17.6%)	14 (12.7%)	11 (6.8%)	17 (16.7%)	13 (20.3%)		
Smoking is a risk factor for cervical cancer						0.980 [0.830-1.160]	*P*=0.810^#^
Yes	15 (44.1%)	41 (37.6%)	59 (36.6%)	34 (33.3%)	27 (42.2%)		
No	19 (55.9%)	68 (62.4%)	102 (63.4%)	68 (66.7%)	37 (57.8%)		
Questions about HPV							
Sexual transmitted						0.520 [0.418-0.648]	*P*<0.001^#^
Yes	33 (97.1%)	97 (88.2%)	132 (82.5%)	73 (71.6%)	35 (54.7%)		
No	1 (2.9%)	13 (11.8%)	28 (17.5%)	29 (28.4%)	29 (45.3%)		
≥ 50% women potentially transmitted						0.744 [0.627-0.883]	*P*<0.001^#^
Yes	15 (44.1%)	51 (46.4%)	65 (40.6%)	32 (31.4%)	14 (21.9%)		
No	19 (55.9%)	59 (53.6%)	95 (59.4%)	70 (68.6%)	50 (78.1%)		
Associstion with cervical cacncer						0.770 [0.652-0.909]	*P*=0.002^#^
Yes	20 (58.8%)	73 (66.4%)	105 (65.6%)	48 (47.1%)	29 (45.3%)		
No	14 (41.2%)	37 (33.6%)	55 (34.4%)	54 (52.9%)	35 (54.7%)		
Effect of HPV vaccination against cancer						0.925 [0.787-1.09]	*P*=0.346^#^
Yes	11 (32.4%)	69 (62.7%)	104 (65.0%)	55 (53.9%)	27 (42.2%)		
No	23 (67.6%)	41 (37.3%)	56 (35.0%)	47 (46.1%)	37 (57.8%)		
Associstion of vulva, anal, and mesopharyngeal cancer						0.962 [0.730-1.270]	*P*=0.782^#^
Yes	3 (8.8%)	11 (10.1%)	17 (10.6%)	6 (5.9%)	7 (10.9%)		
No	31 (91.2%)	98 (89.9%)	143 (89.4%)	96 (94.1%)	57 (89.1%)		

*one patient did not answer question about smoking and association of vulva, anal, and mesopharyngeal cancer

**one patient did not answer question about all HPV item

#Logistic analysis was used

**Table 5:** Cervical cancer diagnosis in patients with different screening intervals

Screening interval	≤ 2 years	> 2 years or never	*P*-value
	*n*=28	*n*=101	
Age			*P*=0.385*
≤ 50 years	13 (46.4%)	37 (36.6%)	
> 50 years	15 (53.6%)	64 (63.4%)	
Histology			*P*=0.058*
SCC	16 (57.1%)	77 (76.2%)	
Others	12 (42.9%)	24 (23.8%)	
Tumor size			*P*=0.155*
≤ 2 cm	11 (39.3%)	25 (24.8%)	
> 2 cm	17 (60.7%)	76 (75.2%)	
Tumor location			*P*<0.005*
Extra-cervix	20 (71.4%)	94 (93.1%)	
Intra-cevical canal	8 (28.6%)	7 (6.9%)	

*Fishier’s exact test was used

## Discussion

Our findings indicated that patients who had never undergone cervical cancer screening or had long intervals between screenings were at a higher risk of advanced cervical cancer than those who underwent regular screenings. Furthermore, patients who underwent screening had low awareness regarding the significance of screening; however, most of them became aware of this after being referred to our hospital. Notably, tumor location within the endocervical canal and non-SCC histology impeded detection of pre-cancerous or early-stage cancer through cervical cancer screening, even with 2-year intervals. These findings suggest the need to reconsider the strategies for screening participation and accuracy.

As shown in Table 1, patients with invasive cancer who had long intervals between cancer screenings or had never undergone cancer screening tended to exhibit advanced stages at the time of diagnosis. This is consistent with previous reports demonstrating that > 50% of patients with cervical cancer never underwent screening or had longer-than-recommended intervals between screenings [14, 15]. Despite extensive efforts in screening programs, screening rates have remained relatively low. A systematic review reported that organized screening was more effective than opportunistic screening [16]. In our region, local municipalities send an invitation letter to eligible people every 2 years, with those who miss the screening receiving the same letter the following year; however, the screening rate remained low at 42.5% in 2022. Therefore, although organized screening may increase the screening rate to some extent, it remained below the satisfactory level in our medical district. Some studies have highlighted the economic–social barriers to cancer screening, and organized screening may reduce inequity [17, 18]. Notably, one study reported that household income was not associated with screening rates [19], as patients are required to pay only 5–10 US dollars out of their pockets owing to subsidies provided by the local government. Furthermore, a USA population-based assessment of cervical cancer screening showed that Asian people were less likely to undergo appropriately timed screening compared with White women [15], which suggests that ethnic culture might affect motivation for screening. Collectively, these findings suggest that increasing the screening rate requires a novel recruitment strategy.

In this study, screening invitation letters contributed to organized screening among approximately 50% of the patients, whereas approximately 20% of patients underwent screening provided at the workplace. However, patients in all age groups were hardly aware of the significance of screening (Table 2). As shown in Table 4, after visiting our hospital, most patients became aware of the lack of clinical symptoms at an early stage and the effectiveness of cancer screening, regardless of their age and screening history. Furthermore, individuals (especially those who were older) had insufficient knowledge regarding HPV, including its transmission route, rate of transmission, effectiveness of vaccination, and association with cervical cancer and other cancers, including vulva, anal, and mesopharyngeal cancers. In the British colorectal cancer screening program, an information booklet about colorectal cancer is sent to eligible people along with the invitation letter. However, 22% of eligible individuals never read this booklet. Furthermore, 63% and 4% of individuals without and with a screening history never read this booklet, respectively [20]. This indicates that simply sending information regarding cervical cancer and the significance of screening may not effectively incentivize eligible individuals to undergo screening. Notably, tailored messages can alter women’s decisions regarding screening participation [21]. Furthermore, primary care physicians who can provide familial messages to patients may remove barriers impeding screening participation [22]. Consequently, if possible, in-person conveyance of messages to patients is crucial for promoting health literacy by utilizing available resources, including the primary doctor, phone calls from the screening organizer, and educational events within the local community.

The age of eligibility for cervical cancer screening is another important factor. Both the American College of Obstetricians and Gynecologists recommendation [23] and the Ministry of Health, Labor and Welfare in Japan recommend the initiation of cervical cancer screening at an age ≥ 20 years every 2 years, as invasive cancer is rare among patients aged < 20 years, with the age-adjusted incidence rate of cervical neoplasm peaking at the age of approximately 40 years. In our study (Table 3), patients who started screening at a younger age were more likely to be diagnosed with a lower CIN stage or early invasive cancer than patients who began screening at an older age. In Australia, where the HPV vaccination program was launched in 2007, a recent simulation based on the declining incidence of cervical neoplasm suggested that the age-adjusted annual incidence of cervical cancer would be as low as four cases in 10,000 women by 2028 with the maintenance of the HPV vaccination and HPV testing program [24]. In our study, none of the patients aged 20–29 years were diagnosed with Stage Ib1–4b. Notably, expanding HPV vaccination coverage could reduce the screening frequency among younger generations within a few decades, even in Japan. However, controversy persists regarding the age at which screening is no longer beneficial for older adults. The US Preventive Services Task Force does not recommend routine screening for women aged ≥ 65 years who have normal Pap smear test results and have adequately adhered to screening [25]. In a Canadian modeling study utilizing registry and survey data, the lifetime risk of cervical cancer in individuals whose screening history was unknown at the age of 70 years could be reduced from 1/158 to 1/1206 by recall for screening [26]. Moreover, Swedish cancer registry data demonstrated that regular screening reduced the cancer risk among individuals aged ≥ 65 years [27]. Considering our findings that older individuals account for a considerable high percentage of patients with invasive cervical cancers, they should be encouraged to undergo screening until the HPV-vaccinated generation reaches old age.

In our study, 28 of the 129 patients with Stage 1b1–4b underwent cervical cancer screening within 2 years. Furthermore, 60.7% of these patients exhibited a tumor of size > 2 cm (Table 5). An almost significant difference was observed in histological findings between patients who had their last screening within 2 years and those with longer intervals. In this study, 9 of the 13 patients who had their last screening within 2 years were aged < 50 years and had non-SCC histology at diagnosis. In patients within this age group, the squamocolumnar junction (SC-junction), from which cervical cancer arises, is usually located outside the cervix and thus allows relatively easy sampling. Notably, cytology has low sensitivity for detecting precancerous lesions of adenocarcinoma [28, 29]. Moreover, an increase has been observed in the worldwide incidence rate of adenocarcinoma against SCC, especially among younger patients [30]. As the HPV-positive rate among patients with adenocarcinoma is approximately 90% [31], the younger generation could be a good candidate for HPV-based screening. However, HPV infection can be transient, and CIN can be regressive in younger people. Therefore, patients with HPV-positive results who have negative cytology should undergo the next HPV test at an adequate interval to avoid unnecessary colposcopy and biopsy, which is further supported by previous findings that a 5-year interval of the HPV test is safer than a 3-year interval of cytology [32]. Another application of the HPV test could be utilized for young patients who maintain fecundity after conization. A study by Bogani et al. [33] demonstrated that the HPV test could detect persistent HPV infection, predicting recurrent cervical dysplasia.

Tumor location was another significant factor in patients diagnosed within 2 years of screening. In this study, six of the eight patients with tumors located in the end-cervical canal and a screening interval of ≤2 years were aged > 50 years. Assuming that the SC-junction migrates toward the deep endocervical canal after menopause, the efficacy of cytological screening in these individuals may be lower than that in younger people [34]. As of 2021, 48 countries have adopted HPV-based screening as the primary method [35]. However, the Catalan Institute investigated the HPV genotype in 10,575 cases, which demonstrated that HPV was detected 87% in squamous carcinoma and 62% in adenocarcinoma [36], and lower HPV positive results were obtained in older patients [37]. Consistent with previous findings [38], we found that a transvaginal ultrasound examination with a Doppler scan can easily detect cervical lesions. Although the cost-benefit balance must be considered, ultrasound examination with cytology screening could be a tailored option for some patients undergoing cancer screening. Finally, physicians must remind patients that screening results may be inaccurate. Therefore, even with a negative screening result, patients with self-reported symptoms should consult a physician to avoid advanced disease from a false-negative result.

Although this study was conducted using single-center data, we believe the results are applicable to communities with low screening rates in Japan and other countries. However, our study also has some limitations, such as the retrospective nature of the study, no review of pathological data by multiple pathologists, and no data collected from women who chose not to undergo screening. We believe that the results of this study will help increase screening rates and eventually decrease cervical cancer mortality.

## Conclusions

Increasing the rate and efficacy of cervical cancer screening is crucial to preventing cervical cancer mortality. In Japan, both cancer screening and HPV vaccination rates are low. Cancer screening is expected to play a crucial role in the detection of cancer precursors in the coming decades. Improving the precision of screening, particularly for non-SCC and endocervical canal tumors, is also important. While cytological screening has significantly reduced cervical cancer incidence and mortality, HPV-based screening may become the future standard. Continuous monitoring of incidence and mortality among different patient groups will help develop more efficient screening strategies.

### Electronic supplementary material

Below is the link to the electronic supplementary material.

Supplementary Material 1

## Data Availability

The data used in this study are available from corresponding author on reasonable request.

## Abbreviations

HPV: Human papillomavirus
SCC: Squamous cell carcinoma
CIN: Cervical intraepithelial neoplasia
SC-junction: Squamocolumnar junction
